# Synergistic influence of mesoporous spinel nickel ferrite on the electrocatalytic activity of nano-structured palladium†

**DOI:** 10.1039/d0ra10944d

**Published:** 2021-03-23

**Authors:** Fariba Kaedi, Zahra Yavari, Ahmad Reza Abbasian, Milad Asmaei, Kagan Kerman, Meissam Noroozifar

**Affiliations:** Department of Chemistry, University of Sistan and Baluchestan Zahedan P.O. Box 98135-674 Iran +98-54-3341-6888 +98-54-3341-6464 z_yavari@chem.usb.ac.ir zahrayavari5@gmail.com; Renewable Energies Research Institute, University of Sistan and Baluchestan Zahedan Iran; Department of Materials Engineering, Faculty of Engineering, University of Sistan and Baluchestan Zahedan Iran; Department of Physical and Environmental Sciences, University of Toronto Scarborough 1265 Military Trail Toronto Ontario M1C 1A4 Canada

## Abstract

Structure and surface area are critical factors for catalysts in fuel cells. Hence, a spinel nickel ferrite mesoporous (SNFM) is prepared *via* the solution combustion technique, an efficient and one-step synthesis. Dynamic X-ray analysis has clarified the structural properties of SNFM. The grain size of SNFM is determined to be ∼11.6 nm. The specific surface area (87.69 m^2^. g^−1^) of SNFM is obtained *via* the Brunauer–Emmett–Teller method. The Barrett–Joyner–Halenda pore size distributions revealed that the size of the mesopores in as-synthesized SNFM mainly falls in the size range of 2–16 nm. Scanning electron microscopy studies showed the regularities involved during porous-structure formation. SNFM is employed as the support for nano-structured palladium (PdNS). Field emission scanning electron microscope studies of PdNS-SNFM showed the deposition of PdNS in cavities and on/in the pores of SNFM. The electrochemical surface area obtained for PdNS-SNFM is about 27 times larger than that of PdNS *via* cyclic voltammetry. The electrochemical studies are utilized to study the features and catalytic performance of PdNS-SNFM in the electro-oxidation of diverse small organic fuels, whereas the electrooxidation of diethylene glycol is reported for first-time.

## Abstract 2

Structure and surface area are critical factors for catalysts in fuel cells.

## Graphical abstract

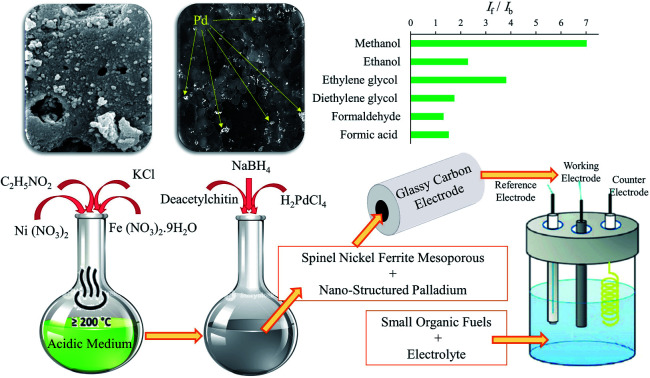

## Introduction

One of the significant factors in fuel cells is modifying the catalytic utility of the supported noble metallic nanoparticles, which relies on the stabilization, distribution, dispersion, and control of particle-size, and on the support options to the necessity of goal reactions.^1^ A practical support has some characteristics, including large surface area, low cost, stability, and high electrical conductivity in the catalysis process.^2^ Hence, for the optimal usage of noble metals, it is essential to fabricate components that have more active sites on the outer surface of the nanocatalyst.^3^ Carbonic supports have a low resistance towards chemical or electrochemical oxidation. Regardless of good conduction (because of carbon structures), these electrocatalysts still are not appropriate because of low usage of noble metal, imperfect mass transportation, and the impermanent stability of carbon-based supports. Accordingly, ceramic materials with durability and high oxidation-resistance are necessary.^4^ Metallic oxides have been employed for the fine dispersion of nanocatalysts.^5,6^ Spinel oxides with the general formula AB_2_O_4_ are a meaningful type of oxides with A^3+^/A^2+^ and B^3+^/B^2+^ redox couples that would allow for good electrocatalytic performance.^7,8^ One of the principal magnetic oxides is nickel ferrite (NiFe_2_O_4_). The Fe and Ni elements of NiFe_2_O_4_ are numerous, environmentally benign, and relatively cheap.^9^ NiFe_2_O_4_, with its superparamagnetic nature and high thermal stability, is often used as a support for magnetic catalysts.^10^ For instance, Gao *et al.* prepared a Pd–NiFe_2_O_4_ catalyst, which significantly increased the catalytic efficiency.^11,12^ Numerous articles have reported various methods of fabricating NiFe_2_O_4_ such as spray pyrolysis,^13^ coprecipitation,^14^ mechanical activation,^15^ hydrothermal methods,^16^ and sol–gel synthesis.^17^ The solution combustion process is a useful and one-step synthesis for the fabrication of metal-oxide catalysts. Porous structure and high crystallization are obtained due to a lot of gases released during the reaction and the exothermicity of the combustion process; in addition, the precursors can improve the chemical structure of the product.^18^

In the last decade, Pd has been considered in the oxidation processes^19^ because it exhibits an impressive electrocatalytic ability, and in contrast to Pt electrocatalysts, Pd catalysts has superb resistance to poisoning from the oxidation of alcohol.^20^ Moreover, the abundance of Pd is about 50 times more than Pt. For this reason, Pd is cheaper than Pt. Consequently, the fabrication of noble metal and mixed oxide on the nanoscale can be the essential factor affecting the efficiency of noble-metal electrocatalysts.^21^ Also, supported nano-particles have been explored in oxidizing conditions, and it has been demonstrated that an excessively thin surface oxide forms before the onset of bulk oxidation. This thin surface oxide is more active in oxidation than corresponding metallic surfaces.^22^

Here, the spinel nickel ferrite mesoporous (SNFM) was synthesized *via* a salt-assisted solution combustion method and characterized *via* a scanning electron microscopy, X-ray diffraction, and Brunauer–Emmett–Teller analysis. The nano-structured palladium (PdNS) in the presence of deacetylchitin as an adhesive agent on the electrode's surface^23^ was placed onto the pores of SNFM. Electrochemical studies were performed to investigate the features and catalytic performance of PdNS-SNFM during the oxidation of diverse small organic fuels (SOFs), including methanol (SOF1), ethanol (SOF2), ethylene glycol (SOF3), diethylene glycol (SOF4), formaldehyde (SOF5), and formic acid (SOF6); whereas diethylene glycol electrooxidation is reported for the first time.

## Experimental

### Chemicals

Nickel(ii) nitrate (Ni(NO_3_)_2_, Carlo Erba), iron(iii) nitrate nonahydrate (Fe(NO_3_)_2_·9H_2_O, May & Baker), deacetylchitin (low molecular weight Fluka), palladium chloride (PdCl_2_ Sigma-Aldrich), and other reagents (Merck) used were of analytical grade.

### Preparation of SNFM and PdNS-SNFM

SNFM was prepared *via* an efficient salt-assisted solution combustion method. The process was as follows: 3.333 g of glycine, 2.908 g of Ni (NO_3_)_2_, 8.080 g Fe (NO_3_)_2_·9H_2_O and, 1.49 g potassium chloride were mixed in a known amount of acidic water (pH = 4), respectively. The resulting mixture was continually kept on a heater to facilitate the combustion reaction. The as-prepared sediments were heated in deionized water to eliminate solvable by-products. The sediments were separated *via* an external magnetic field, washed with deionized water, and eventually dried at 85 °C for 5 h. To fabricate PdNS-SNFM, 3 ml deacetylchitin solution (0.018 g deacetylchitin + 100 ml 1% volumetric acetic acid aqueous solution) was added to 5 mg PdCl_2_ and 20 μl HCl 37%. In a second container, 3 mg SNFM was added to 0.5 ml deacetylchitin solution. After ultrasound, two as-prepared mixtures were added together and stirred on a rotary apparatus at 200 rpm for a full day. Then, 100 μl of NaBH_4_ solution (100 mg ml^−1^) was added and again stirred at the same speed for 4 h.

### Instrumentation and electrochemical tests

For the physical description of samples, X-ray diffraction (XRD) was applied using Bruker, Advanced D8. The infrared spectra of the as-synthesized samples were recorded in the range of 400–4000 cm^−1^ on a Fourier transform infrared (FTIR) spectrometer (Bruker, TENSOR II). Microstructure evaluations were carried out *via* the scanning electron microscopy (SEM, KYKY EM3900) technique. The field emission scanning electron microscopy (FESEM) analyses were done on an SAMX electron microscope (MIRA3 TESCAN). The adsorption/desorption isotherms of SNFM were obtained *via* the N_2_ adsorption technique using a BELSORP-mini II instrument. The specific surface areas and the pore size distribution were assessed *via* the Brunauer–Emmett–Teller (BET) technique and the Barrett–Joyner–Halenda (BJH) concept, respectively. Standard *t*-curve calculated from eqn (1) by converting the adsorption isotherm of the vertical axis to the *t* value.1
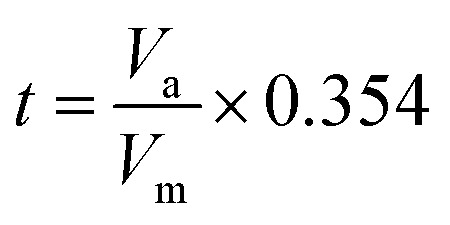
where *t* is the thickness of the adsorption layer, *V*_m_ is the gas volume at the mono-layer coverage, and *V*_a_ is the whole gas volume adsorbed at standard conditions (*T* = 273.15 K and *P* = 101.3 kPa).

The electrochemical tests were performed by an SAMA 500 electroanalyzer (SAMA Center, Iran) in a cell containing: a Pt (as the auxiliary electrode) electrode, Hg/HgO (as the reference electrodes) electrode, and glassy carbon (GC, as the working electrode) electrode with 3.14 × 10^−6^ m^2^ surface area. The working electrode was prepared as follows: (1) mechanical polishing, (2) electrochemical activation in an acidic medium by an anodic and cathodic sweep, (3) spraying 10 μl of catalytic composite on the surface electrode, and (4) solvent evaporation of the composite and the creation of a catalytic layer on the surface electrode, which were denoted as GC/PdNS-SNFM and GC/PdNS electrodes.

## Results and discussions

### Evaluation of SNFM and PdNS-SNFM

**Fig. 1:**
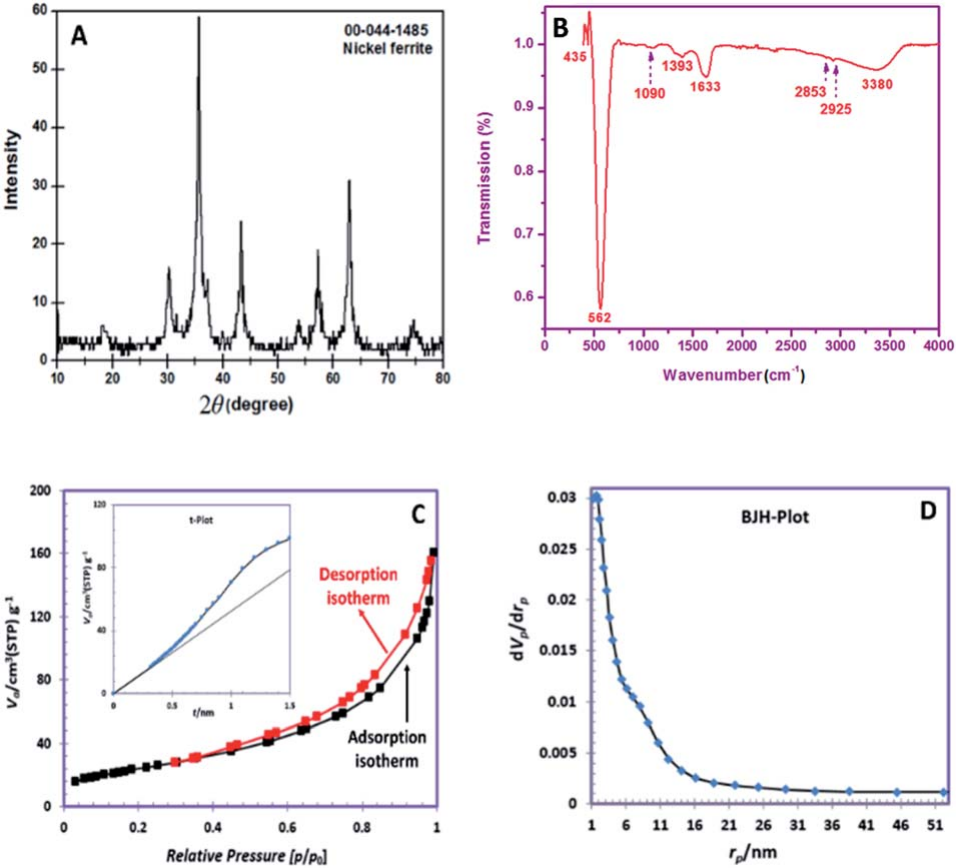
(A). XRD pattern, (B) FTIR spectrum, (C) nitrogen adsorption and desorption isotherms (inset t-plot), (D) BJH pore size distributions.

The XRD pattern of the obtained powder is shown in Fig. 1A. The observed peaks are in good agreement with the pure cubic spinel NiFe_2_O_4_ phase (JCPDS No. 044-1485). The peaks appeared at 2*θ* degrees of 18.43, 30.23, 35.59, 37.14, 43.32, 53.80, 57.22, 62.89 and 74.68 can be well-indexed to the (111), (220), (311), (222), (400), (422), (511), (440) and (533) planes of NiFe_2_O_4_ phase, respectively. The grain size of NiFe_2_O_4_ obtained *via* the Debye–Scherrer's relation was ∼11.6 nm.^24^

Fig. 1B depicts the FTIR spectrum of SNFM powders. The broad band at around 3380 cm^−1^ is responsible for the presence of the O–H group.^25^ The weak bands at 2926 and 2853 cm^−1^ can be assigned to C–H asymmetric and C–H symmetric stretching frequencies, respectively.^26^ The band around 1633 cm^−1^ correspond to the vibration of the N–H bond.^27^ The band at 1393 cm^−1^ corresponds to NO^3−^ ions.^28^ The observed weak band at 1090 cm^−1^ is due to C=O stretching with ring stretching.^29^ The bands at 562 and 435 cm^−1^ indicate the cubic spinel structure formation of NiFe_2_O_4_. The band at 435 cm^−1^ proves metal–oxygen stretching in the octahedral position. The intense peak at 562 cm^−1^ is correlated to the metal-oxygen stretching tetrahedral band.^30^

The N_2_ adsorption/desorption isotherms of the obtained SNFM powder is presented in Fig. 1C. The hysteresis profile is detected to form IV class with H3 hysteresis, which suggests the presence of mesopores.^31^ Also, the t-plot of SNFM powders is shown in the inset of Fig. 1C. The t-plot depicts a sharp straight line that begins from the origin point but becomes smoother after some point. Therefore, SNFM powders belong to mesoporous materials. Likewise, SNFM powder exhibits a specific surface area and total pore volume of 87.69 m^2^ g^−1^ and 0.2377 cm^3^ g^−1^, respectively. The BJH pore dimension distributions are also depicted in Fig. 1D, which shows that the size of mesopores in the as-synthesized SNFM mainly falls in the range of 2–16 nm. The presence of slit-shaped pores and panel-shaped particles generates the H3 hysteresis. Due to non-rigid particle aggregates, the isotherms revealing type H3 do not limit adsorption at high *P*/*P*_0_.^31^ Consequently, it seems that the brittle agglomerations of porous particles result in IV isotherms with H3 hysteresis.

**Fig. 2:**
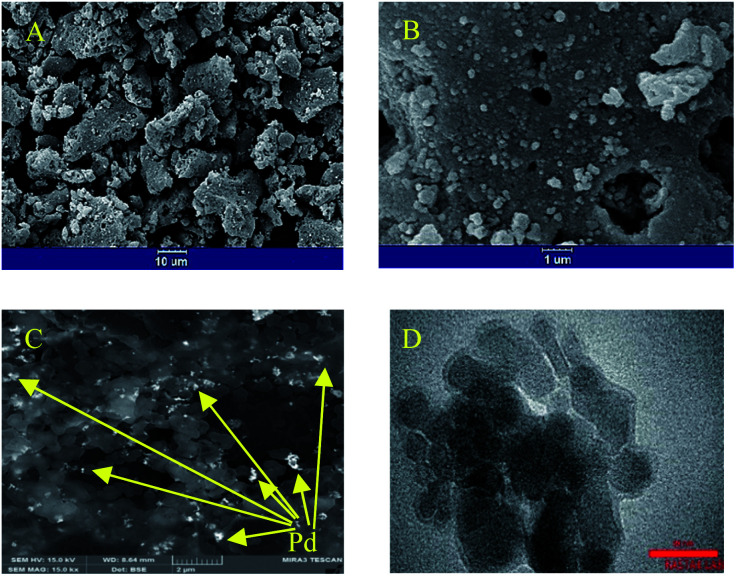
The SEM images with (A) 10, and (B) 1 μm scales for SNFM; (C) FESEM image of PdNS-SNFM, with 100 μm scale, and (D) TEM micrograph of PdNS-SNFM with 50 μm scale.

In the SEM micrograph with a lower magnification (Fig. 2A), a porous and spongy structure of SNFM is observed. In another image with higher magnification (Fig. 2B), the effect of combustion is more apparent. The combustion created tiny cavities in SNFM powders. Also, it can be seen that agglomerates are formed by assembling nanoparticles. Therefore, Pd can be inserted on the surface and in the cavities of SNFM.

To consider the morphology of PdNS-SNFM, FESEM images are shown in Fig. 2C. It is clear that the holes were occupied with PdNS (brighter points) in the SNFM structure (darker places). Therefore, the formation of PdNS into holes and onto the surface of SNFM was demonstrated. Nevertheless, there are unfilled cavities in PdNS-SNFM.

Such cavities improve the fuel storage on the surface catalyst for electrooxidation. The good dispersion of PdNS on SNFM is marked.

Fig. 2D displays the TEM micrograph for PdNS-SNFM in 50 nm scale. It confirmed that the palladium particles formed in nano-size with diameter range of 10–20 nm. As in the FESEM image, the unfilled holes in the TEM image are visible.

### Electrocatalytic performance

**Fig. 3:**
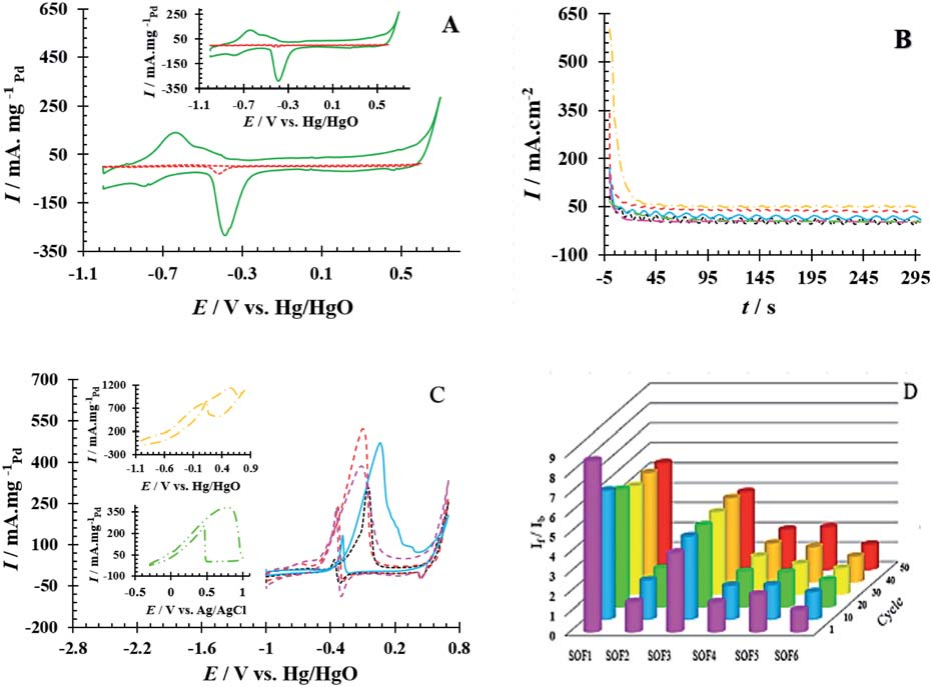
(A) The CV curves for GCE/PdNS (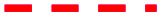) and GCE/PdNS-SNFM (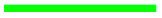) electrodes with the Pd loading of 0.31 mg cm^−2^ in 1 M NaOH (inset: after 80 cycles), (B) The CA curves at *E* = 0.1 V, (C) CV curves (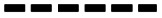) 0.80 M SOF1, (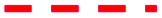) 0.55 M SOF2, (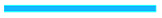) 0.55 M SOF3, (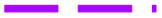) 0.34 M SOF4, (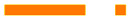) 0.43 M SOF_5_ and (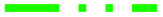) 0.85 M SOF6 electroxidation on the GCE/PdNS-SNFM electrode, and (D) bar chart of *I*_f_/*I*_b_ stability.

The electrochemical performance of PdNS and PdNS-SNFM was considered *via* cyclic voltammetry. Fig. 3A shows CVs for PdNS and PdNS-SNFM after 1 run (inset: 80 run) with a Pd loading of 0.31 mg cm^−2^. The voltammetric features of the modified electrodes reveal the typical behaviour of palladium. The observed peaks for both electrodes in the different regions are attributed to several electrochemical processes. The forward sweep directed an electrooxidation process on the electrode surface, resulting in the creation of Pd oxides (+0.3 to +0.7 V *vs.* Hg/HgO) and hydrogen desorption (−0.9 to −0.4 V *vs.* Hg/HgO), while the reverse sweep reflected the reduction of the Pd oxides (−0.2 to −0.5 V *vs.* Hg/HgO) and hydrogen adsorption (−0.6 to −0.9 V *vs.* Hg/HgO).^32^ In 2005, Ketteler *et al.* found that the Pd surface oxidation proceeds by the formation of stable and metastable structures,^33^ such as PdO and PdOH in positive voltages.^34^ The accumulation of oxygenated compounds affects the adsorption of hydrocarbons on the surface of the working electrode by changing the electrocatalyst surface potential. Respectively, the potential region of −0.2 to +0.4 V *vs.* Hg/HgO was related to the double layer on the working electrode.

The electrochemical surface area (EAS_H_/EAS_Pd_) is important to define electrocatalyst efficiency.^35^ EAS_H_ and EAS_Pd_ are respectively estimated over the surface below the peak adsorption/desorption hydrogen (*Q*_H_) and the surface below the curve of Pd–O reduction (*Q*_Pd_).^36,37^ The durability of the as-synthesized electrocatalysts was examined by applying potential cycling in an alkaline medium. Comparisons of EAS_H_ and EAS_Pd_ at the beginning and end of the test were used to analyze the loss EAS rate. *Q*_H_ (1^th^ cycle = 104.18 and 80^th^ cycle = 103.28 mC cm^−2^) and *Q*_Pd_ (1^th^ cycle = 127.39 and 80^th^ cycle = 122.36 mC cm^−2^) were obtained on GCE/PdNS-SNFM, while *Q*_H_ (1th cycle = 4.00 and 80^th^ cycle = 2.66 mC cm^−2^) and *Q*_Pd_ (1^th^ cycle = 7.04 and 80^th^ cycle = 4.01 mC cm^−2^) were on GCE/PdNS. This data exhibited that EAS_H_ and EAS_Pd_ for PdNS-SNFM were considerably improved relative to PdNS. The dispersion of PdNS was dropped after durability for modified electrodes, representing decrease in EAS_H_ and EAS_Pd_ due to sintering and dissolution of PdNS. The loss percentages were 33.55 and 9.75 for PdNS and 1.26 and 3.94 for PdNS-SNFM on EAS_H_ and EAS_Pd_, respectively. These data are presented in Table S1.† A comparison showed that using a mesoporous support enhanced the dispersion and electrochemical surface area of PdNS. PdNS-SNFM had superior distribution and durability compared to PdNS. SNFM with three-dimensional porous construction can be sufficient for inhibiting sintering, agglomeration and, dissolution of PdNS. Therefore, the stability of the electrocatalyst can be improved through SNFM. The size and dispersion of PdNS were better due to the creation of PdNS on and into the pores of SNFM as the support.

Also, the stability of PdNS-SNFM and PdNS were studied *via* chronoamperometry (CA). Fig. 3B shows the chronoamperograms of SOF1–SOF6 oxidation on the PdNS-SNFM catalysts at 0.1 V potential for 300 s. In the beginning, *I vs. t* curves meaningfully dropped (about 10 first seconds). Next, the current density was approximately stable owing to the falling number of active sites on the electrocatalyst to substitute the SOF molecules. Such enhanced stability is explained *via* the improved creation of OH groups on the SNFM surface.^38^ The reaction among such groups and middle species may increase the durability of PdNS-SNFM. As shown in Fig. 3B, SOF5 has a greater current density (637.01 mA cm^2^) compared to the other SOF.

The number of fuel molecules per electrocatalyst surface per second is equal to the turnover number (TON). This factor determines the current in steady-state for a given electrooxidation process. The number of fuel monolayers that are electrooxidized per unit time is known as the turnover frequency (TOF). These two factors can be estimated according to ref. 39. The electrochemical results from Fig. 3B were presented in Table S2.†

The electrooxidation reaction of SOFs on PdNS-SNFM was evaluated utilizing the CV technique. Fig. 3C shows the CV curves related to SOF1–SOF6 electrooxidation on GCE/PdNS-SNFM with 0.05 V s^−1^. The peak that appeared in the forward sweep (*I*_f_) is ascribed to the oxidation of the adsorbed species including SOF. In contrast, the second peak in the backward sweep (*I*_b_) is responsible for the oxidation of created intermediate components that were not completely oxidized in the forward scan.^39^ A likely pathway for the SOF oxidation reaction is expressed in Table 1.^40–44^

**Table 1:** The likely pathway for the SOF oxidation reaction

SOF1 (ref. 40)	SOF2 (ref. 41)	SOF3 (ref. 42)	SOF5 (ref. 43)	SOF6 (ref. 44)
Pd + CH_3_OH → Pd−CH_3_OH_ads_	Pd + CH_3_CH_2_OH → Pd−CH_3_CH_2_OH_ads_	Pd + (CH_2_OH)_2_ → Pd-(CH_2_OH)_2 ads_	Pd + H_2_O → Pd–OH_ads_ + H+ + e^−^	The general reactions
				HCOOH + Pd → HCOO−Pd + H^+^ + e^−^
				HCOOH + Pd → Pd−CO + H_2_O
Pd−CH_3_OH_ads_ + OH^−^ → Pd−CH_3_O_ads_ + H_2_O + e^−^	Pd−CH_3_CH_2_OH_ads_ + 3OH^−^ → 3H_2_O + Pd−CH_3 ads_ + 3e^−^	Pd–(CH_2_OH)_2 ads_+ 4OH^−^ → Pd–(HCO)_2 ads_+ 4H_2_O + 4e^−^	Pd + HCHO_sol_ → Pd−HCHO_ads_	Formic acid electrooxidation reaction in the direct pathway
				HCOO−Pd → Pd−H + CO_2_
				Pd−H → Pd + H^+^ + e^−^
Pd−CH_3_O_ads_ + OH^−^ → H_2_O + Pd−CH_2_O_ads_ + e^−^	Pd−CH_3_CO_ads_ + Pd−OH_ads_ → Pd + Pd−CH_3_COOH	Pd−HCO_2 ads_ + 4OH^−^ → Pd−HCOO_2 ads_ + 2H_2_O + 4e^−^	Pd−HCHO_ads_ → Pd–CHO_ads_ + H^+^ + e^−^	Formic acid electrooxidation reaction in the indirect pathway
				Pd + H_2_O → Pd−OH + H^+^ + e^−^
				Pd−CO + Pd−OH → Pd + CO_2_ + H^+^ + e^−^
Pd−CH_2_O_ads_ + OH^−^ → Pd−CHO_ads_ + H_2_O + e^−^	Pd−CH_3_COOH + OH^−^ → Pd + CH_3_COO^−^ + H_2_O	Pd–HCOO_ads_ + e^−^ → Pd–CO_ads_ + OH^−^	Pd–CHO_ads_ → Pd–CO_ads_ + Pd–OH_ads_ + H^+^ + e^−^	Net reaction
Pd−CHO_ads_ + OH^−^ → Pd−CO_ads_ + 4H_2_O + e^−^		Pd–HCO_ads_ + e^−^ → Pd–CO_ads_ + OH^−^	Pd–CO_ads_ + Pd–OH_ads_ → Pd + Pd–COOH_ads_	HCOOH → CO_2_ + 2H^+^ + 2e^−^
Pd−CO_ads_ +2OH^−^ → Pd + CO_2_ + H_2_O + 2e^−^		Pd−OH^−^ → Pd + OH_ads_ + e^−^	Pd + Pd–COOH_ads_ → 2Pd + CO_2_ + H^+^ + e^−^	
		Pd–CO_ads_ + Pd + OH_ads_ → 2Pd + CO_2_ + H^+^ + e^−^		

The stability of the *I*_f_/*I*_b_ ratio during 50 cycles is shown in the bar chart in Fig. 3D. It is responsible for the earlier elimination of intermediates on the PdNS surface due to the higher surface area. It indicates that the synergistic properties of SNFM on PdNS performance enhance the electrocatalytic ability of PdNS-SNFM as a multi-functional electrocatalyst in SOF electrooxidation. Structural oxygens in SNFM are the active species to eliminate the intermediates of SOF electrooxidation. Support containing interface metal like NiFe_2_O_4_ is a promoter agent for the dehydrogenation as the initial step of oxidation of small organic fuels due to their multi-oxidative state *via* the orienting C–H bond; because it can be desirable to create the cycling redox among the high and low valences.

The Ni^3+^/Ni^2+^ and Fe^3+^/Fe^2+^ redox couples exhibit good electrocatalytic performance. As stated before, the thin surface oxide formed is more active in oxidation than corresponding metallic surfaces.^22^ Presumably, the surface oxygen of ferrite activated the oxidation process. On the other hand, the porous network of NiFe_2_O_4_ prevents PdNS aggregation. The synergistic influence can be because of the earlier removal of intermediates due to increased surface area and weakened agglomeration of PdNS, thus decreasing the resistance of mass transfer to store small organic fuels into the holes of NiFe_2_O_4_ ceramic support.

### Adsorption isotherms on the PdNS-SNFM surface

**Fig. 4:**
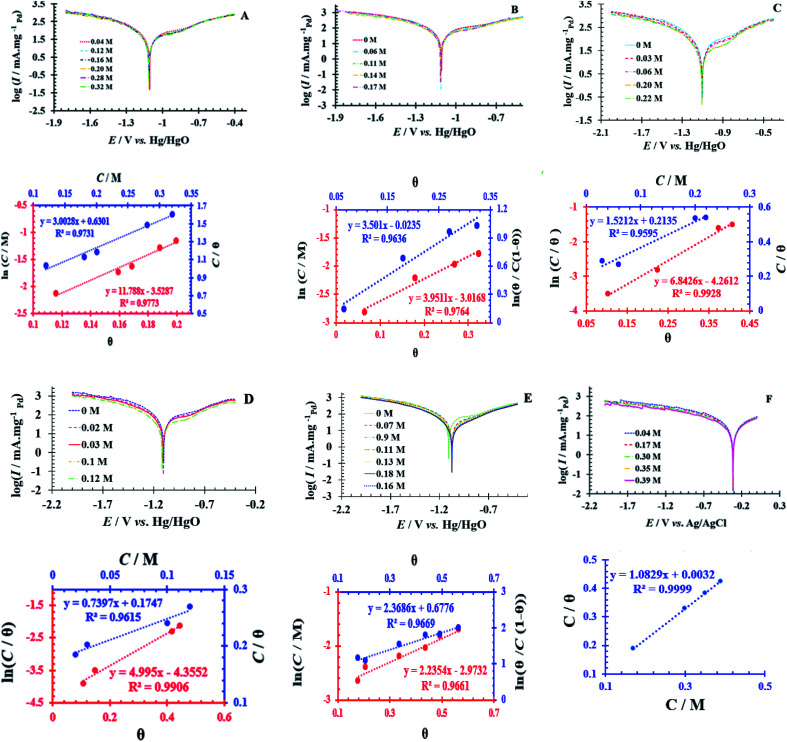
Tafel curves at different concentrations of SOF1–SOF6 and the resulting charts: the linear outcomes fitted according Temkin (ln *C vs. θ*), Langmuir (*C*/*θ vs. C*) and Frumkin (ln *θ*/*C* (1 − *θ*) *vs. θ*) adsorption isotherms on the surface of the PdNS-SNFM catalyst.

Simultaneously, with the SOF adsorption process on the electrode surface, a competition happens among the adsorbed hydrogen on the modified electrode surface and SOF in the bulk solution. The adsorption is assessed *via* isotherms^45,46^ (See Fig. 4 and Table 2). The initial stage in SOF oxidation is the adsorption of SOF molecules as obtained by Temkin isotherm,^47^ which assumes that the interaction among the adsorbed species and that the as mentioned exists a competition among adsorbed species (SOFs and H_2_).^46,48^

**Table 2:** The value of correlation coefficients (*R*^2^) plots of isotherms for each fuel

	SOF1	SOF2	SOF3	SOF4	SOF5	SOF6
Temkin: ln *C* = −ln *K* + *aθ*	0.977	0.976	0.992	0.990	0.966	0.638
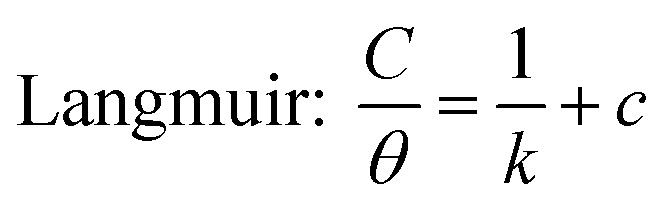	0.973	0.864	0.959	0.961	0.495	0.999
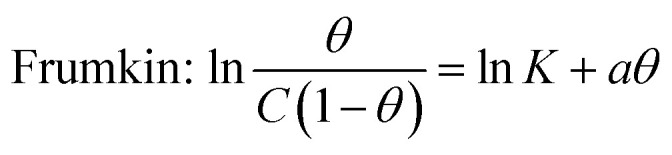	0.758	0.963	0.466	0.810	0.966	0.549
*C* = SOF concentration; *K*_ads_ = equilibrium constant; *θ* = surface coverage; 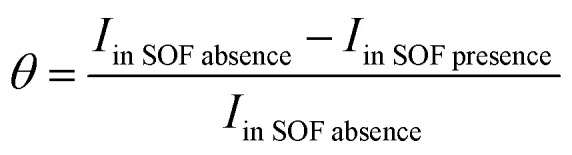						

The accessible sites on PdNS were particular for H_2_ adsorption during enhancing SOF concentration. Consequently, the exchange current was decreased. The degree of surface coverage (*θ*) determines the outcome of species competition in the adsorption. Hence, the range of potential for linear polarization the gradient of Langmuir isotherm displays the formation of the SOF mono-layer on the electrode surface. The SOF was adsorbed on the active sites of PdNS-SNFM without any interaction among them.^46^ The slope amounts of Langmuir isotherm for SOF1–SOF6 were not equal to the unit. Hence, it is clear that there are the significant interactions among absorbed species.

## Conclusion

SNFM was prepared by the solution combustion technique. SNFM exhibited a specific surface area of 87.69 m^2^. g^−1^. Therefore, it was employed for the formation of PdNS into cavities and onto the surface of SNFM. The value of EASH for PdNS-SNFM was about 27 times more than PdNS. The catalytic performance was investigated for SOF electrooxidation on the PdNS-SNFM catalyst by the electrochemical studies. In an overview, the PdNS-SNFM activity was decreased relative to the electro-oxidation of different fuels as follows: SOF5 > SOF2 > SOF3 > SOF4 > SOF6 > SOF1. It is concluded that the synergistic influence of SNFM on the catalytic activity of PdNS can be due to the improvement of the surface area, distribution, and stability of PdNS. In addition, it enlarged the ability of removing the compounds by blocking the active sites of the catalyst for the electro-oxidation of SOFs with the help of surface oxygens, and the creation of the cycling redox between the high and low chemical valents of the intermediate metals such as nickel and iron.

## Conflicts of interest

There are no conflicts to declare.

## Supplementary Material

RA-011-D0RA10944D-s001
