# Supplementary material for: Synergistic influence of mesoporous spinel nickel ferrite on the electrocatalytic activity of nano-structured palladium
Source: RSC Adv. 2021 Mar 23;11(20):11813–20. doi: 10.1039/d0ra10944d (PMC8696967; doi:10.1039/d0ra10944d)
Supplement: RA-011-D0RA10944D-s001 [file RA-011-D0RA10944D-s001.pdf]

Electronic Supplementary Material (ESI) for RSC Advances.
This journal is © The Royal Society of Chemistry 2021

Supporting information

Synergistic Influence of Spinel Nickel Ferrite Mesoporous on Electrocatalytic Activity of Nano-Structured Palladium

Fariba Kaedi[1], Zahra Yavari*[1,2], Ahmad Reza Abbasian[3], Milad Asmaei[3], Kagan Kerman[4], Meissam Noroozifar*[4]

1. *Department of Chemistry, University of Sistan and Baluchestan, Zahedan, P.O. Box 98135-674, Iran*
2. *Renewable Energies Research Institute, University of Sistan and Baluchestan, Zahedan, Iran*
3. *Department of Materials Engineering, Faculty of Engineering, University of Sistan and Baluchestan, Zahedan, Iran*
4. *Department of Physical and Environmental Sciences, University of Toronto Scarborough1265 Military Trail, Toronto, Ontario, M1C 1A4, Canada*

*Corresponding Authors: Tel.: +98-54-3341-6464; Fax: +98-54-3341-6888.

z_yavari@chem.usb.ac.ir; ORCID: 0000-0003-1049-0448

ahmadabbassian@gmail.com; ORCID: 0000-0002-8560-289X

m.noroozifar@utoronto.ca; ORCID:0000-0001-7997-0461

Solution	Electrode	GCE/PdNS		GCE/PdNS-SNFM	
		1th cycle	80th cycle	1th cycle	80th cycle
1 M NaOH	$Q_H{}^{a}$ (mC.cm^{-2})	4.00	2.67	104.18	103.28
	$EAS_H{}^{b}$ (m^2.g^{-1})	3.07	2.05	82.18	79.32
	$D_{Pd}{}^{c} \times 10^{-4}$	22.02	14.71	590.12	569.59
	% $loss_{ADT_H}{}^{d}$	33.22		1.26	
	Q_{pd} (mC.cm^{-2})	7.05	4.01	127.39	122.36
	$EAS_{Pd}{}^{e}$ (m^2.g^{-1})	3.19	2.88	101.46	79.32
	% $loss_{ADT_{Pd}}{}^{f}$	9.72		3.94	

a. $Q_H = \frac{Q'_H + Q''_H}{2}$ {electrochemically desorption (Q'_H) and adsorption (Q''_H) of H_2 molecules on the Pd sites}

b. $EAS_H = \frac{Q_H}{k \times l_{Pd}}$ {k = 0.420 mC.cm^{-2} and l_{Pd} = 0.310mg.cm^{-2}}

c. $D_{Pd} = \frac{EAS_H}{\frac{1}{M_{Pd}} \times (N_A \times 4\pi \times r_{Pd}^2)}$ {M_{Pd} = 106.42 g.mol^{-1}, $N_A = 6.02 \times 10^{23}$ mol^{-1} and $r_{Pd} = 0.14 \times 10^{-9}$ m}

d. $\%loss_{ADT_H} = \frac{\left(EAS_{H_{1^{th}cycle}} - EAS_{H_{150^{th}cycle}}\right)}{EAS_{H_{1^{th}cycle}}} \times 100$

e. $EAS_{Pd} = \frac{Q_{Pd}}{s \times l_{Pd}}$ {s = 0.405 mC.cm^{-2} and l_{Pd} = 0.310 mg.cm^{-2}}

f. $\%loss_{ADT_{Pd}} = \frac{\left(EAS_{Pd_{1^{th}cycle}} - EAS_{Pd_{150^{th}cycle}}\right)}{EAS_{Pd_{1^{th}cycle}}} \times 100$

Table S1. The calculated results from CVs in absence of SOFs.

CO stripping and EIS studies for PdNS and PdNS-SNFM

The linear sweep voltammograms (LSVs) for PdNS and PdNS-SNFM were provided at -0.3 to 0.1 V and 0.05 V.s^{-1} (Fig.S1 A) as CO stripping. The current for CO stripping peak on PdNS-SNFM (= 47.65 mA.mg$^{-1}{}_{Pd}$) was more than PdNS (= 33.23 mA.mg$^{-1}{}_{Pd}$). In addition to, the CO peak potential for PdNS (= -0.10 V) was shifted to negative values for PdNS-SNFM (= -0.11 V).

The $Q_{CO\ ads}$ were 29.07 and 20.19 mC.cm^{-2} for PdNS-SNFM and PdNS, respectively. They can because of the CO removal on the Pd surface over the larger surface area. The roughness factor (r_f) and the mass-specific surface area (A) were calculated from LSVs using [S1]. r_f and A was calculated for PdNS and PdNS-SNFM, (48.92 and 155.12) (69.21 and 223.27 m^2.g^{-1}). A comparison proved that the SNFM existence in the role of support increases the surface area of PdNS.

Fig. S1 B illustrates the Nyquist curves of GCE/PdNS and GCE/PdNS-SNFM electrodes. The Nyquist curves detect a charge transfer process on the GCE/PdNS and GCE/PdNS-SNFM electrodes. The obtained data exhibited the resistances of charge transfer (R_{ct}) on GCE/PdNS, and GCE/PdNS-SNFM electrodes were 60.22 and 16.24 Ω, respectively. On the other hands, R_{ct} for PdNS is decreased in the presence of SNFM. The exchange current (I_0) for the electrodes were calculated by [S2]:

$$I_0 = \frac{R \times T}{n \times F \times R_{CT}} \tag{1}$$

The parameters are R gas constant, T absolute temperature, n the exchanged electrons number and F Faraday constant The I_0 on GCE/PdNS and GCE/PdNS-SNFM was about 0.426 and 1.580 mA, correspondingly. It can be due to the higher rate of charge transfer.

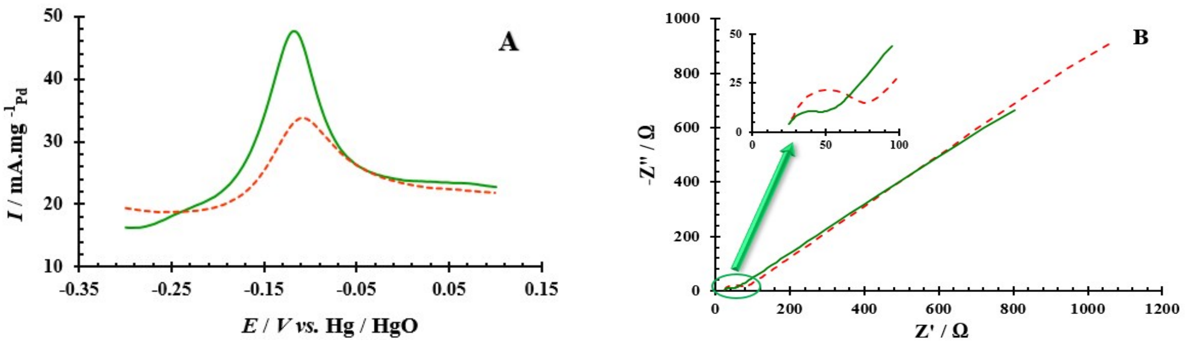

Fig. S1 (A) CO stripping of in NaOH 1 M, and (B) Nyquist plots for (- - -) GCE/PdNS and (——) GCE/PdNS-SNFM electrodes in the solution of 0.005 M $[Fe(CN)_6]^{3-/4-}$prepared in 0.1 M KCl over a frequency region of 1 ×10^{-1} to 1 ×10^{5} Hz at 0.30 V.

Table S2. The calculated results from CAs on GC/PdNS-SNFM.

The data of this work are comparable to other presented catalysts in article for electrooxidation reaction of different organic compounds as shown in Table S3 [S3-S9]. Up to Table S3, the I_f in SOF5 oxidation was much higher and peak potential (E_f) SOF4 oxidation is lower than other fuels. Furthermore, the onset potential (E_{onset}) SOF4 and SOF5 oxidation were more negative than the

other fuels. In order to determine electrocatalyst tolerance to accumulate carbonaceous intermediate, the ratio of I_f/I_b was calculated.

	$I_{(t = 0\ s)}$ ($mA.mg^{-1}{}_{Pd}$)	$J_{(t = 300\ s)}$ ($mA.cm^{-2}$)
0.80 M SOF1	257.72	9.87
0.55 M SOF2	1070.04	33.11
0.55 M SOF3	535.13	12.12
0.34 M SOF4	489.35	2.23
0.43 M SOF5	1887.50	51.89
0.85 M SOF6	210.72	2.67

Table S3. The electrochemical parameter as estimated from CV curves for electrooxidation reaction of SOF on PdNS-PdNS and another catalyst.

Electrocatalyst	Fuels	Concentration (M)	Potential Range (V)	Reference Electrode	E_{onset} (V)	E_f (V)	I_f	E_b (V)	I_b	I_f / I_b	Ref.
Pd/RGO	SOF1	1	-0.8 to +0.4	Ag/AgCl	-0.34	-0.19	388 mA.mg^{-1}	-0.34	138.57 mA.cm^{-2}	2.8	[S3]
Pd-PNMSS	SOF1	0.20	-1 to +0.7	Hg/HgO	-0.76	-0.09	97.48 mA.cm^{-2}	-0.38	12.53 mA.cm-2	7.78	[S2]
	SOF2	20	-1 to +0.7	Hg/HgO	-0.85	-0.09	157.90 mA.cm-2	-0.38	40.19 mA.cm-2	3.93	
Pd-Ag-NS	SOF2	1	-0.7 to +0.2	Hg/HgO	-0.72	-0.20	2.34 mA.cm^{-2}	-0.28	1.02 mA.cm^{-2}	2.28	[S4]
Pd_1Au_1NSFs[a]	SOF3	0.50	-0.8 to +0.4	SCE	-	-0.08	355 mA.mg^{-1}	-	-	-	[S5]
Pt@Pd NCs	SOF3	0.50	-0.8 to +0.4	SCE	0.026	-1.4	58.4 mA.cm^{-2}	-	-	-	[S6]
Fe/FeZSM-5CPE	SOF5	0.13	0 to +1	SCE	-	0.52	19.08 mA.cm^{-2}	0.25	-	-	[S7]
Pt/CNx[b]	SOF5	1	0 to +1	Ag/AgCl	0.15	0.72	301 mA.mg^{-1}	-	485.48 mA.cm^{-2}	0.62	[S8]
	SOF6	1	0 to +1.1	Ag/AgCl	0.00	0.73	493 mA.mg^{-1}	-	-	1.15	
$Pd_{10}Bi_1$/C	SOF6	0.50	-0.2 to +1	Ag/AgCl	0.08	0.00	9.20 mA.cm^{-2}	-	-	-	[S9]
PdNS-PdNS	SOF1	0.80	-1 to +0.7	Hg/HgO	-0.97	-0.05	308.45 mA.mg^{-1}	-0.35	43.91 mA.mg^{-1}	7.02	In this work
	SOF2	0.55			-0.94	-0.10	520.4 mA.mg^{-1}	-0.33	228.68 mA.mg^{-1}	2.28	
	SOF3	0.55			-0.98	+0.05	469.85 mA.mg^{-1}	-0.29	123.27 mA.mg^{-1}	3.81	
	SOF4	0.34			-0.98	-0.11	386.21 mA.mg^{-1}	-0.35	221.10 mA.mg^{-1}	1.75	
	SOF5	0.43			-1	+0.50	1130.29 mA.mg^{-1}	-0.11	859.00 mA.mg^{-1}	1.31	
	SOF6	0.85	-0.3 to +1	Ag/AgCl	-0.23	+0.80	377.78 mA.mg^{-1}	-0.40	248.32 mA.mg^{-1}	1.52	

a. PdAu nanosnowflakes.
b. Platinum-carbon nitride

Calculation of electrochemical factors for SOFs oxidation on PdNS-SNFM surface

The SOFs electrooxidation on GCE/PdNS-SNFM electrode was a consideration under the difference conditions; whereas the electrochemical data as valued from CVs curves were reported as Table S4. In the test of SOF concentration (Fig. S2 A-F), with increasing SOF1-SOF6 concentration to 0.95, 1.37, 0.42, 0.59, 0.43 and 0.35 M, respectively, the current was increased. Nevertheless, any significant increase doesn't observe in SOF concentrations higher than mention concentration for every SOF. This is owing to the occupancy of active sites on the electrode surface. The logarithm I versus logarithm [SOF] is designed to calculate the order of reaction [S10]. The order of reaction gotten from the slope of the line for forward and backward scanning in SOF1-SOF6 oxidation (Inset Fig. S2 A-F) was recorded in Table S4. Base on Table S4, reaction order for the anodic sweep in SOF2 was more than other fuels and after that were SOF4, SOF3, SOF1, SOF6, and SOF5 respectively and in the anodic sweep, SOF5 was higher and after that were SOF1, SOF4, SOF3, SOF2, and SOF6 respectively. Consequently, measure SOF2 oxidation and intermediate of SOF5 oxidation was higher than other fuels.

The temperature effect on the forward and backward currents in different temperatures between 20 and 60 ˚C was showed in Fig. S3 A-F. The forward and backward currents were enlarged with the increasing temperature. The relevance between Arrhenius and appropriate linear fit ln I vs. T^{-1} delivered the activation energy (E_a) for anodic and cathodic sweeps [S11]. The plots of ln I vs. T^{-1} are shown in Inset Fig. S3 A-F. The values of E_a for SOF1-SOF6 electrooxidation at the anodic and cathodic sweep were calculated and recorded in Table S4. As it is clear, the Ea reduction order is as follows in the cathodic scan: SOF2>SOF1>SOF5>SOF4>SOF3>SOF6 and in the anodic scan: SOF3>SOF4>SOF2>SOF1>SOF5>SOF6. Thus, the oxidation of SOF6 and oxidation intermediates produced in SOF6 oxidation was faster than other fuels. As well, with accelerating

in the scanning, current was improved at forward and backward scanning (Fig. S4 A-F). By slop of the plots the I_f versus the square root of the sweep rate ($\nu^{1/2}$) and the E_p versus ln (v) (See Inset Fig. S4 A-F), electron transfer (α), and diffusion (D) coefficients were calculated according to [S3] and reported in Table S4. Electron transfer in SOF3 with $\alpha = 0.99$ and diffusion in SOF1 was more than in comparing with other SOF. The linear correlation among E_p and ln (v) and I_f and $\nu^{1/2}$ proved the SOF1-SOF6 oxidation is the irreversible charge transfer phenomenon, and the transport of SOF molecules from bulk solution to the electrode surface is as the rate determining step for total reaction, respectively.

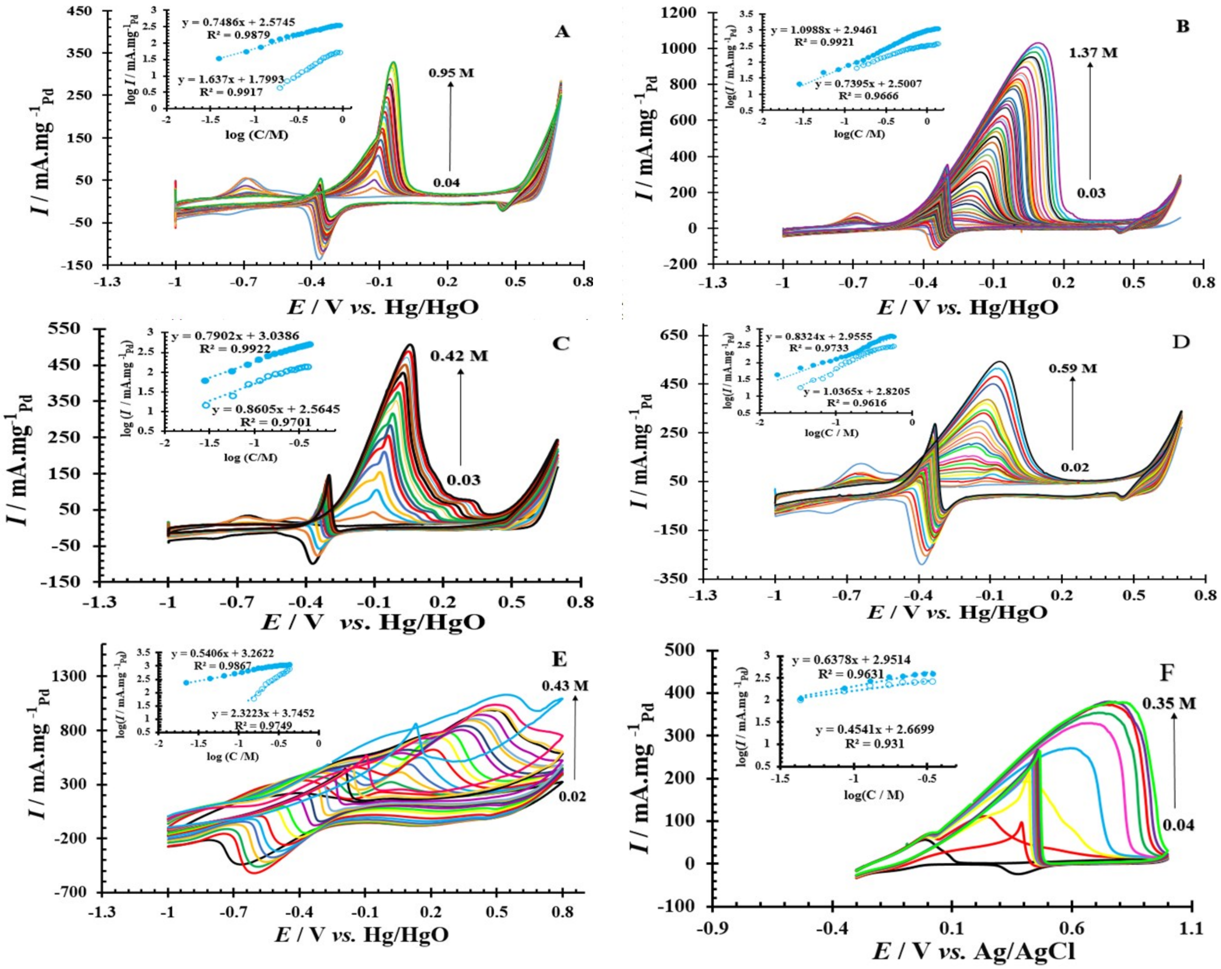

Fig. S2. CVs of SOF1-SOF6 oxidation (A-F) in various concentration (Inset: The plot of log I_f in (●) anodic and (○) cathodic sweep *vs.* log *C*) on GCE/PdNS-SNFM electrode in 1 M NaOH for SOF1-SOF5 and 0.1 M H_2SO_4 for SOF6 at 0.05 $V.s^{-1}$ sweep rate.

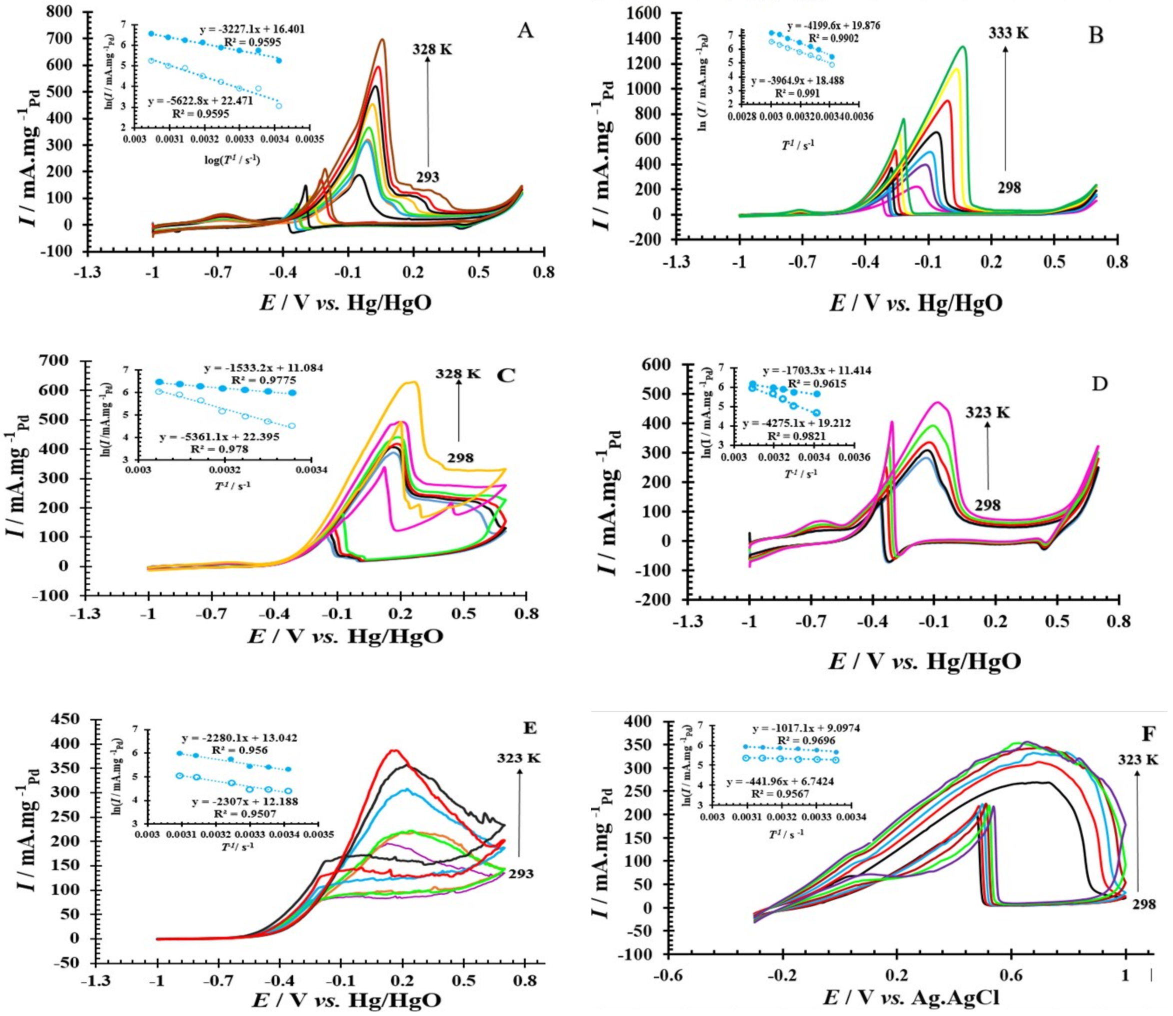

Fig. S3. CVs of SOF1-SOF6 electrooxidation (A-F) at various temperatures. (Inset: plot of ln I_f in (●) anodic and (○) cathodic sweep vs. T^{-1} on GCE/PdNS-SNFM electrode in 1 M NaOH for SOF1-SOF5 and 0.1 M H_2SO_4 for SOF6.

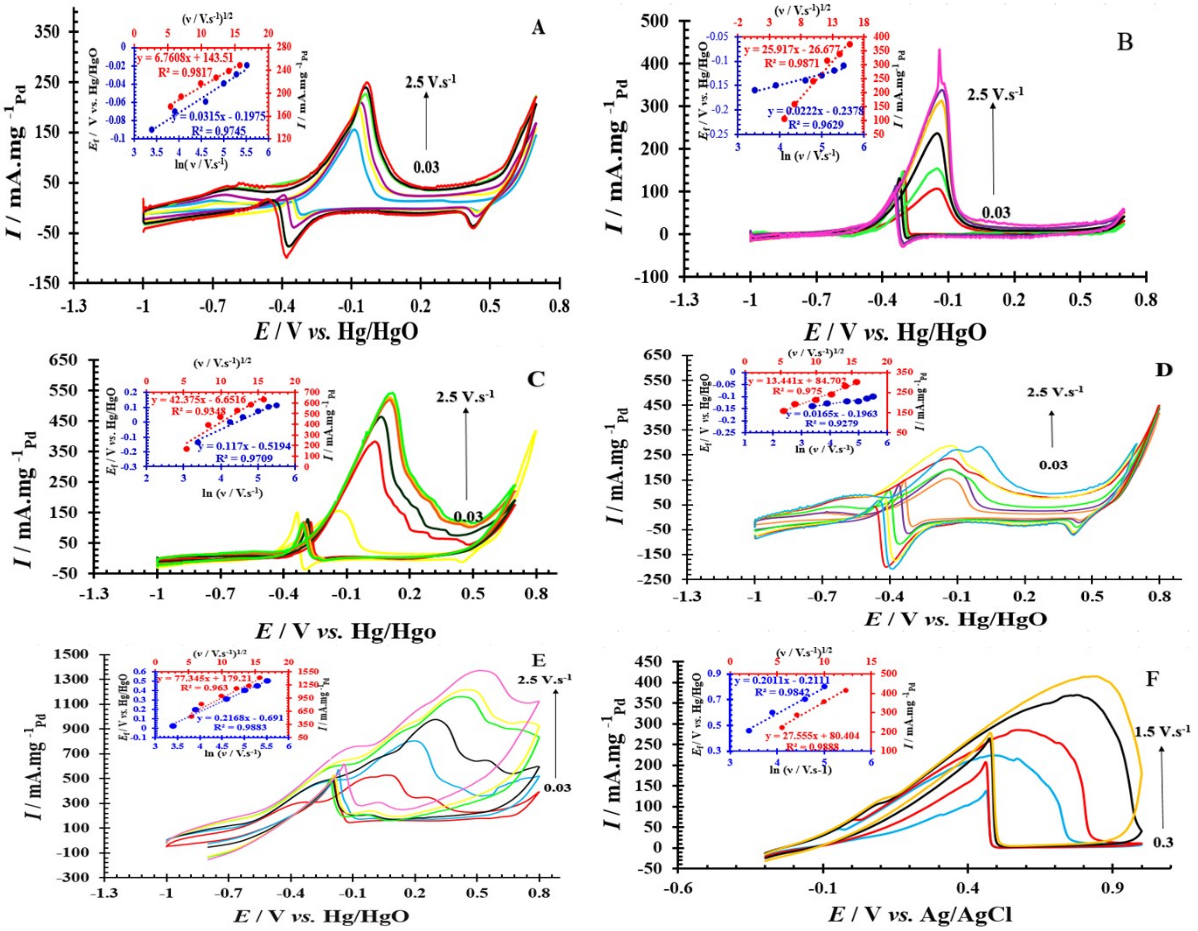

Fig. S4. CVs of SOF1-SOF6 electrooxidation (A-F) at various sweep rate (Inset: The plots of E_f vs. ln υ and I_f vs. $\nu^{1/2}$) on the GCE/PdNS-SNFM electrode in 1 M NaOH for SOF1-SOF5 and 0.1 M H_2SO_4 for SOF6.

Table S4. The investigation results effect of analyte concentration, temperature and scan rate on GCE/PdNS-SNFM electrode in liquid fuels electroxidation.

Fuel	Reaction Order[a]		E_a[b] (kJ.mol^{-1})		δE_f / δlnυ	δJ_f / $\delta υ^{1/2}$	α[c]	C (mol/cm^3)	n*	D[d*] (cm^2/s)
	Forward sweep	Backward sweep	Forward sweep	Backward sweep						
SOF1	0.74 (R^2 = 0.987)	1.63 (R^2 = 0.991)	26.83 (s^+ = -3227.1 & R^2 = 0.959)	29.87 (s = -5622.8 & R^2 = 0.959)	0.03 (R^2 = 0.974)	6.76 (R^2 = 0.981)	0.86	800	6	4.23×10^{-2}
SOF2	1.09 (R^2 = 0.992)	0.73 (R^2 = 0.966)	34.91 (s = -4199.6 & R^2 = 0.990)	32.96 (s = -3964.9 & R^2 = 0.991)	0.02 (R^2 = 0.962)	0.03 (R^2 = 0.987)	0.90	550	12	2.10×10^{-2}
SOF3	0.79 (R^2 = 0.992)	0.86 (R^2 = 0.970)	12.74 (s = -1533.2 & R^2 = 0.977)	44.57 (s = -5361.1 & R^2 = 0.978)	8.29 (R^2 = 0.970)	42.37 (R^2 = 0.934)	0.99	550	10	6.60×10^{-8}
SOF4	0.83 (R^2 = 0.973)	1.03 (R^2 = 0.961)	14.16 (s = -1703.3 & R^2 = 0.961)	35.54 (s = -4275.1 & R^2 = 0.982)	0.01 (R^2 = 0.927)	13.44 (R^2 = 0.975)	0.86	340	18	3.43×10^{-9}
SOF5	0.53 (R^2 = 0.996)	2.96 (R^2 = 0.959)	18.95 (s = -2280.1 & R^2 = 0.956)	19.18 (s = -2307.0 & R^2 = 0.950)	0.21 (R^2 = 0.988)	77.34 (R^2 = 0.963)	0.97	430	4	4.59×10^{-5}
SOF6	0.63 (R^2 = 0.963)	0.45 (R^2 = 0.931)	8.45 (s = -1017.1 & R^2 = 0.969)	3.67 (s = -441.9 & R^2 = 0.956)	0.20 (R^2 = 0.984)	27.55 (R^2 = 0.988)	0.94	850	2	1.53×10^{-6}

a. $\log I = n \log C + B$ {n= reaction order, C = concentration and B = interception}

b. $$E_a = -\left(\frac{\delta lnI_p}{\delta\left(\frac{1}{T}\right)} \times R\right)$$ {E_a = activation energy and R = gas constant}

c. $$\alpha = 1 - \frac{R \times T}{\frac{\delta E_p}{\delta(ln υ)} \times n \times F}$$ {α = electron transfer coefficient, $υ$ = scan rate, R = gas constant, T = 293 K, F = Faraday constant and n = electrons number transferred during oxidation of one mol analyte}

d. $$D = \frac{\left(\frac{\delta J_P}{\delta υ^{1/2}}\right)^2 \times R \times T}{2.46 \times 10^{-7} \times (n \times F)^3 \times \alpha \times C^2}$$ {D = diffusion coefficient and C = concentration}

* If the oxidation reaction be complete (to produce CO_2), and not partial oxidation.

\# R-squared in linear fitting

\+ Slop of fitted line

References

[S1] Zhang, L.Y..; Zhao, Zh. L.; Li, Ch.M. Formic acid-reduced ultrasmall Pd nanocrystals on graphene to provide superior electocatalytic activity and stability toward formic acid oxidation. *Nano Energy* 2015, 11, 71-77.

[S2]. Kaed, F.; Yavari, Z..; Noroozifar, M..; Saravani, H.. Promoted electrocatalytic ability of the Pd on doped Pt in NiO-MgO solid solution toward methanol and ethanol oxidation. *J. Electroanal. Chem*. 2018, 827, 204-212.

[S3]. Yang, F.; Zhang, B.; Dong, S.; Tang, Y.; Hou, L.; Chen, Zh.; Li, Z.; Yang, W.; Xu, Ch.; Wang, M.; Li, Y. Silica nanosphere supported palladium nanoparticles encapsulated with graphene: High-performance electrocatalysts for methanol oxidation reaction. *Appl. Surf. Sci.* 2018, 452, 11–18.

[S4]. Peng, Ch.; Hu. Y.; Liu, M.; Zheng, Y. Hollow raspberry-like PdAg alloy nanospheres: High electrocatalytic activity for ethanol oxidation in alkaline media. *J. Power Sources* 2015, 278, 69-75.

[S5]. Zh. Z. Yang.; L. Liu.; A.J. Wang.; Yuan, J..; Feng, J.J.; Q. Qing Xu. Simple wet-chemical strategy for large-scaled synthesis of snowflake-like PdAu alloy nanostructures as effective electrocatalysts of ethanol and ethylene glycol oxidation. *J. Int. J. Hydrogen Energy* 2017, 42, 2034-2044.

[S6]. Li, D. N.; He, Y. M.; Feng, J. J.; Zhang, Q. L.; Zhang, L.; Wu, L.; Wang, A. J. Facile synthesis of prickly platinum-palladium core-shell nanocrystals and their boosted electrocatalytic activity towards polyhydric alcohols oxidation and hydrogen evolution. *J. Colloid Interface Sci.* 2018, 514, 476-483.

[S7]. Abrishamkar, M..; Barootkoob, M.. Electrooxidation of formaldehyde as a fuel for fuel cells using Fe^{2+}-nano-zeolite modified carbon paste electrode. *Int. J. Hydrogen Energy* 2017, 42, 23821-23825.

[S8]. Sadhukhan, M.; Kundu, M. K.; Bhowmik, T.; Barman, S. Highly dispersed platinum nanoparticles on graphitic carbon nitride: A highly active and durable electrocatalyst for oxidation of methanol, formic acid and formaldehyde. *Int. J. Hydrogen Energy* 2017, 42, 9371-9383.

[S9]. Yang. S.; Yang. J.; Chung, Y. Pd-Bi bimetallic catalysts including polyvinylpyrrolidone surfactant inducing excellent formic acid oxidation reaction and direct formic acid fuel cell performance. *Int. J. Hydrogen Energy* 2017, 42, 17211-17220.

[S10]. Wang, E. D.; Xu, J. B.; Zhao, T. S. Density functional theory studies of the structure sensitivity of ethanol oxidation on palladium surfaces. *J. Phys. Chem. C* 2010, 114, 10489-10497.

[S11]. Haghnegahdar, S.; Noroozifar, M. Deposition of PdPtAu nanoparticles on hollow nanospheres of Fe_3O_4 as a new catalyst for methanol electrooxidation: Application in direct methanol fuel cell. *Electroanalysis* 2017, 29, 1-11.
